# Experimental Investigation of Failure Behaviors of CFRP–Al Lap Joints with Various Configurations Under High- and Low-Temperature Conditions

**DOI:** 10.3390/ma18153467

**Published:** 2025-07-24

**Authors:** Mingzhen Wang, Qiaosheng Huang, Qingfeng Duan, Wentao Yang, Yue Cui, Hongqiang Lyu

**Affiliations:** 1 College of Aerospace Engineering, Nanjing University of Aeronautics and Astronautics, Nanjing 210016, China; 2 China Special Aircraft Research Institute, Jingmen 448035, China; 3 China Energy Science and Technology Research Institute Co., Ltd., Wuhan 430070, China; 4 School of Mechanical and Aerospace Engineering, Nanyang Technological University, Singapore 639798, Singapore

**Keywords:** bonded–bolted joint, lap joints, tensile failure, high and low temperatures, aircraft structures

## Abstract

The failure behaviors of CFR–aluminum lap joints with diverse configurations through quasi-static tensile tests were conducted at −40 °C, 25 °C, and 80 °C. Four specimen types were examined: CFRP–aluminum alloy two-bolt single-lap joints (TBSL), two-bolt double-lap joints (TBDL), two-bolt bonded–bolted hybrid single-lap joints (BBSL), and two-bolt bonded–bolted hybrid double-lap joints (BBDL). The analysis reveals that double-lap joints possess a markedly higher strength than single-lap joints. The ultimate loads of the TBSL (single-lap joints) at temperatures of −40 °C and 25 °C are 29.5% and 26.20% lower, respectively, than those of the TBDL (double-lap joints). Similarly, the ultimate loads of the BBSL (hybrid single-lap joints) at −40 °C, 25 °C, and 80 °C are 19.8%, 31.66%, and 40.05% lower, respectively, compared to the corresponding data of the TBDL. In bolted–bonded hybrid connections, the adhesive layer enhances the joint’s overall stiffness but exhibits significant temperature dependence. At room and low temperatures, the ultimate loads of the BBDL are 46.97 kN at −40 °C and 50.30 kN at 25 °C, which are significantly higher than those of the TBDL (42.24 kN and 44.63 kN, respectively). However, at high temperatures, the load–displacement curves of the BBDL and TBDL are nearly identical. This suggests that the adhesive layers are unable to provide a sufficient shear-bearing capacity due to their low modulus at elevated temperatures. This research provides valuable insights for designing composite–metal connections in aircraft structures, highlighting the impacts of different joint configurations and temperature conditions on failure modes and load-bearing capacities.

## 1. Introduction

Fiber-reinforced polymer (FRP) composites, with their superior specific strength, specific stiffness, and designability, are widely used in engineering structures such as aircraft, ships, and vehicles [1,2]. However, their anisotropy, non-homogeneity, and brittleness pose significant challenges for connecting FRP composites to metal structures. The inevitable hole-making process in these connections disrupts fiber continuity in the composite plates, reducing the structural cross-sectional area. When loaded, the stress distribution around the holes becomes highly complex. Unlike metals, FRP composites remain mostly linear-elastic until failure without local yielding, preventing stress redistribution around the holes and intensifying the concentration of stress. Their multi-component and non-uniform nature also makes them prone to failure at local sites of maximum stress, minimum strength, or inherent weakness, causing complex and unpredictable bolted-joint failure modes. Numerous factors affect the strength and lifespan of bolted joints in FRP–metal connections. These include the composite ply orientation, joint configuration, geometry, manufacturing process, and load type, as well as environmental factors. Additionally, the mismatch in thermal expansion coefficients and stiffness between metals and composites can lead to asymmetric deformation and non-uniform failure under thermal or mechanical loads, further compromising joint performance. Given the complexity, the design of connections between composites and metals is crucial for the broad application of composites in engineering. Many scholars have carried out extensive experimental research and numerical simulations on the failure analysis of composite connection structures [3,4,5,6,7,8,9,10,11,12,13,14,15,16,17,18], as well as in-depth analyses of the bearing capacity and failure modes.

Composite–metal connections can be single-lap (single-shear bolted) or double-lap (double-shear bolted). Single-lap connections can be further subdivided into single-bolt single-lap, double-bolt single-lap, and multi-bolt single-lap types. Under a tensile load, single-lap structures experience eccentric loads. This produces moments in bolts, causing out-of-plane joint bending and compressive deformation around holes, known as the secondary bending effect. Ekh and Schön et al. [3] used finite element analysis to study the impact of secondary bending on composite joint strength prediction. The results showed that secondary bending increases the contact area between fasteners and hole edges, reduces bearing stresses, and boosts bearing strength. Cao et al. [6] examined damage evolution and failure patterns in single-lap thin-layered composite bolted connections under quasi-static loads. They found that thin-layered composites have an extra inhibitory effect on bearing partition delamination growth under secondary bending loads. Zhang et al. [10] investigated the effects of environmental aging on the static and fatigue mechanical behavior of single-lap Al–CFRP joints. Using cohesive zone elements [19,20,21], they predicted damage evolution in the bonding region under hygrothermal conditions and studied the fatigue failure characteristics of single-shear joints under different environmental and load conditions. Cao et al. [22] developed a micro-scale model to simulate progressive damage and failure in double-lap thin-ply laminated composite bolted connections, accurately capturing local bolt-hole crushing in the late loading stage. Moreover, even in single-lap joints, the use of double or multi-bolts can, to a certain extent, mitigate the secondary bending effect. Zhao et al. [4], through tensile failure tests and numerical simulations, compared the secondary bending effect in single-shear and double-shear bolted connections. They discovered that single-lap joints exhibit secondary bending, while double-lap joints show no obvious secondary bending due to structural symmetry. In addition, multi-bolt configurations can help mitigate the secondary bending effect in single-lap joints. Mehrabian et al. [23] performed experimental tests, demonstrating that employing multi-bolt configurations can reduce the secondary bending effect.

Another important type of composite–metal connection is the bonded–bolted hybrid connection [7,9,12,13,14,16,24], which combines the advantages of adhesive and bolted connections. Many scholars have made significant progress in this field. Jiang et al. [24] experimentally studied the effects of different ply sequences and lap modes on the performance of bonded, bolted, and hybrid woven CFRP joints, offering key data for joint design. Mehrabian et al. [7], using advanced 3D DIC technology, precisely measured the strain fields of bolted and hybrid bonded–bolted joints in woven carbon fiber epoxy composites, aiding in joint performance evaluation. Mohapatra et al. [16] employed a 3D damage evolution model based on the modified Hashin criteria to study the damage initiation and propagation in woven GFRP fiber plate joints, providing a theoretical tool for predicting joint life and reliability. Ulus [9] evaluated the mechanical properties of basalt fiber-reinforced polymer composite hybrid bolted–bonded joints under varying temperatures, offering insights into the impacts of temperature on joint performance. Xiang Sheng et al. [12,13] combined non-destructive monitoring and numerical simulations to study the fatigue behavior of GFRP hybrid bonded–bolted joints under shear loads and analyzed failure behaviors under static shear, supporting the reliability-based design of GFRP joints. Yokozeki et al. [14] conducted large-scale experiments to explore the mechanical behavior of hybrid joints composed of preloaded bolts and adhesive bonds, advancing our understanding of this novel connection form. Existing studies have systematically investigated metal–composite connections, including single-lap, double-lap, and pure bolted or bonded–bolted hybrid connections. However, most research has focused on room temperature conditions, with limited attention to high- and low-temperature environments. The effects of different connection methods on the failure modes and connection strength of metal–composite structures under extreme temperatures remain under-researched.

In this paper, four types of specimens were prepared: CFRP–aluminum alloy two-bolt single-lap joints (TBSL), two-bolt double-lap joints (TBDL), two-bolt bonded–bolted hybrid single-lap joints (BBSL), and two-bolt bonded–bolted hybrid double-lap joints (BBDL). The bearing capacity and failure modes of these joints were studied at −40 °C, 25 °C (room temperature, RM), and 80 °C. Additionally, two extra specimens, namely open-hole laminates (OHT) and one-bolt double-lap specimens (OBDL), were prepared for comparative analysis in tensile tests.

## 2. Specimen Preparation and Experimental Testing

### 2.1. Specimen Preparation

This study performed quasi-static tensile tests on TBSL, TBDL, BBSL, and BBDL specimens at three typical temperatures: −40 °C, 25 °C, and 80 °C. Comparable tests on open-hole laminates (OHT) and one-bolt double-lap specimens (OBDL) were also performed for contrast. The composite laminates use ACTECH^®^1203/GW7011/38 (Zhonghang Composites, Beijing, China) medium-temperature curing epoxy carbon fiber prepregs, with a layup of [45_2_/0_20_/45_2_], 24 plies, and a total thickness of about 4.8 mm. The metal connection plates are made of a 2024–T4 aluminum alloy, with a thickness of 5 mm. The fasteners are self-locking bolts (HB—103) and nuts (GB1337) made of ML30Cr—MaSiA structural steel, with a diameter D = 5 mm. The adhesive is 3M™ DP490™ Epoxy.

**Figure 1:**
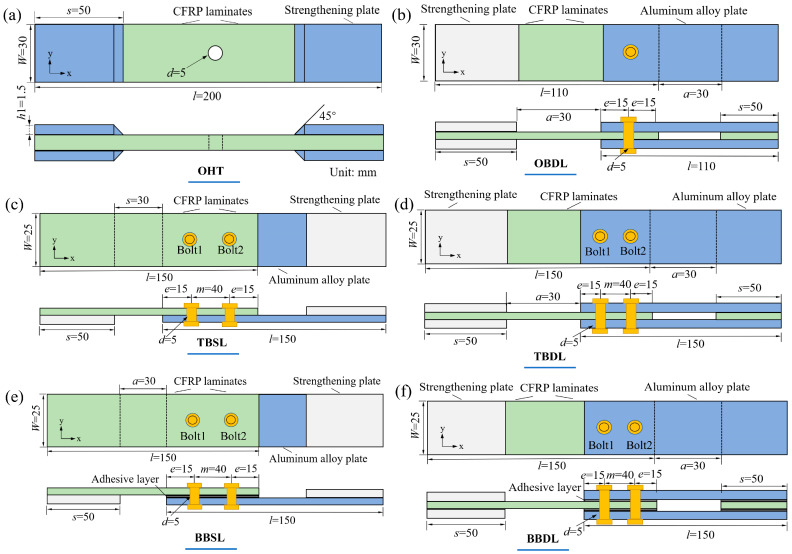
Geometric dimensions of the composite–aluminum joints: (**a**) OHT; (**b**) ODBL; (**c**) TBSL; (**d**) TBDL; (**e**) BBSL; (**f**) BBDL.

According to ASTM D5961 [25], the composite laminates and aluminum plates have the same dimensions: width W = 25 mm, length L = 150 mm, end distance e = 15 mm, fastener hole pitch m = 40 mm, and adhesive thickness 0.2 mm. The dimensions of the single- and double-lap hybrid specimens are shown in Figure 1. For pure bolted connections, the materials and dimensions are the same as the hybrid ones, but without the central adhesive layer. The specimens’ designations are provided in Table 1.

Composite components were prepared using a manual layup and autoclave molding process. A layer of polytetrafluoroethylene (PTFE) cloth was first placed on a clean marble mold to facilitate de-molding. Over this, a breathable release fabric was laid to aid in air removal. Pre-cut prepreg sheets were then positioned on top of the breathable fabric. Throughout the layup, a rubber roller was used to eliminate air bubbles and ensure proper adhesion, preventing the introduction of defects. Every four layers, a vacuum operation was performed. During this step, the prepreg was sequentially covered with a breathable release fabric, a perforated isolation film, and an absorbent adhesive layer. The entire assembly was then sealed with a vacuum bag. After completing the layup, the vacuum was drawn for 10 min to remove any entrapped air. Finally, the assembly was sealed with high-temperature-resistant sealing tape and vacuum bags, placed in an autoclave, and cured under heat and pressure to achieve the desired composite structure. The pressure–time and temperature–time curves for composite material molding are conducted using the process provided by the supplier.

**Figure 2:**
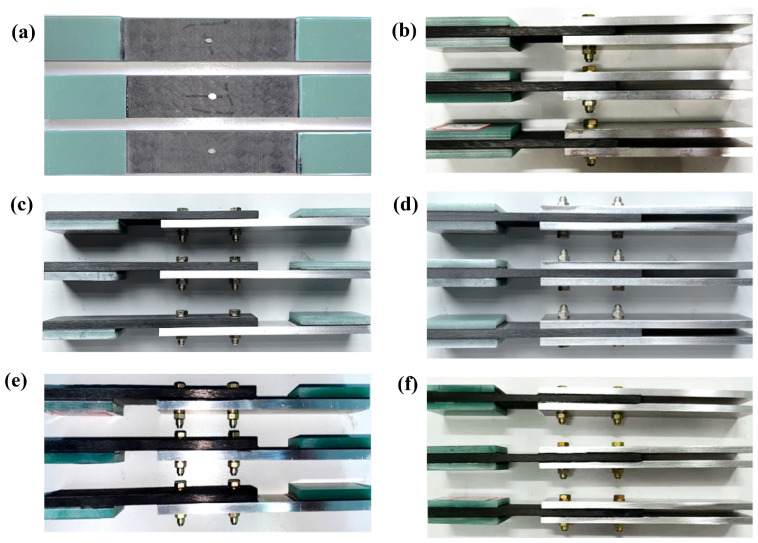
Open-hole laminate and composite–aluminum connection specimens: (**a**) OHT, (**b**) OBDL, (**c**) TBSL, (**d**) TBDL, (**e**) BBSL, and (**f**) BBDL.

**Figure 3:**
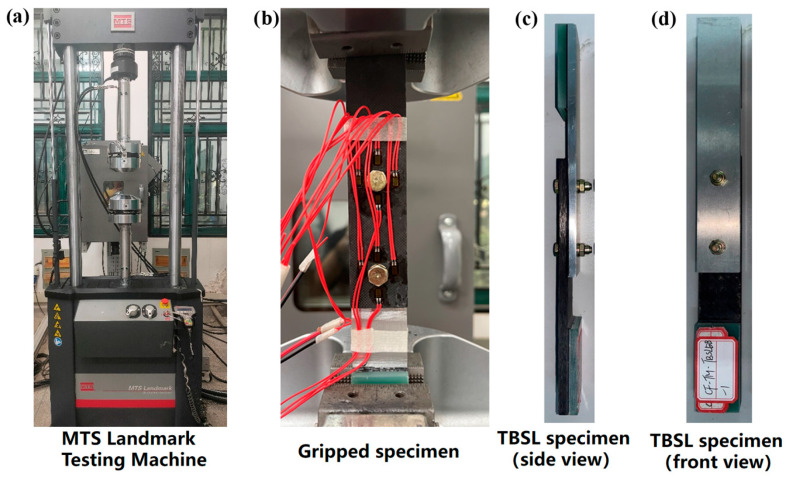
Test equipment and specimen installation: (**a**) MTS Landmark universal testing machine; (**b**) gripped specimen; (**c**) side view of two-bolt single-lap specimen; and (**d**) front view of two-bolt single-lap specimen.

After demolding upon curing completion, the composite panel was bonded with doublers and machined to the dimensions specified in Figure 2. Subsequently, through-holes were prepared for the composite–aluminum alloy bonded specimens using a diamond-coated drill bit, with the hole tolerance maintained at grade H8. Finally, the machined panels were assembled with fasteners to fabricate the composite–aluminum alloy bonded joint specimens, as depicted in Figure 3.

### 2.2. Experimental Testing

Quasi-static tensile failure tests of the composite specimens were performed using an MTS universal testing machine. The tests took place in a high- and low-temperature chamber, where the temperature was regulated to expose the specimens to the desired testing temperatures.

A displacement-controlled loading method was employed at a rate of 1 mm/min while continuously recording the load–displacement curves. Table 1 lists the testing conditions, with each test repeated three times to ensure data validity and reliability. The failure modes of the composite specimens were photographed and analyzed. Figure 3 illustrates the assembly configurations and geometric dimensions for all test scenarios. The testing setup was an MTS universal testing machine (Figure 3a). For TBSL, Figure 3b shows the specimen mounted on the fixture, and actual photos of the TBSL specimen are displayed in Figure 3c,d.

## 3. Experimental Results

### 3.1. Tensile Failure Results of Open-Hole Laminates

**Figure 4:**
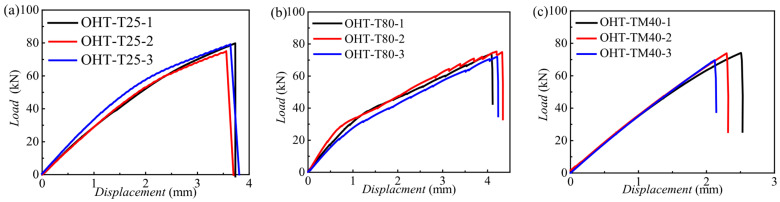
Load–displacement curves of CFRP open-hole laminates at three typical temperatures: (**a**) load–displacement curves for CFRP open-hole laminate specimens (OHT) at 25 °C; (**b**) load–displacement curves for CFRP open-hole laminate specimens (OHT) at −40 °C; (**c**) load–displacement curves for CFRP open-hole laminate specimens (OHT) at 80 °C.

**Figure 5:**
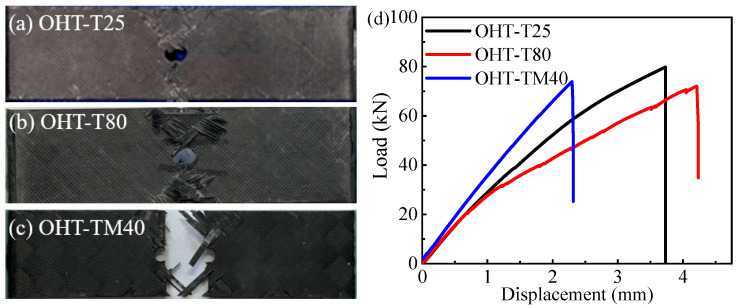
Tensile failure modes of CFRP open-hole laminates at (**a**) 25 °C, (**b**) 80 °C, and (**c**) −40 °C; (**d**) load–displacement comparison curves of the three temperatures for open-hole specimens.

In accordance with ASTM D5766 [26], tensile experiments were conducted on open-hole tension (OHT) specimens, with detailed records of the force–displacement curves and failure modes. Figure 4 shows the load–displacement curves for three groups of CFRP open-hole laminate specimens (OHT) at 25 °C, −40 °C, and 80 °C, with consistent results across tests. Figure 5 displays the tensile failure modes and load–displacement comparison curves of the three temperatures. It is evident that the specimen holes exhibited significant tensile deformation with the net-tension failure mode. The 45° layup showed a fan-shaped failure mode, and the failure modes were generally similar across the temperatures.

At low temperatures, the open-hole laminates displayed greater tensile stiffness but smaller failure displacement compared to room temperature and high-temperature environments. This is attributed to the softening of the composite resin matrix at high temperatures, which reduces the tensile modulus, and its hardening at low temperatures, which increases the tensile modulus. As shown in Figure 5d, the ultimate tensile load of the open-hole laminates was highest at room temperature, followed by at high temperatures, and was lowest at low temperatures. This can be explained by the fact that the failure of open-hole laminates is primarily governed by net-tension failure. The ultimate tensile strength of the open-hole laminates at different temperatures is closely related to the tensile strength of the composite itself.

### 3.2. Tensile Failure Results of OBDL Specimens

**Figure 6:**
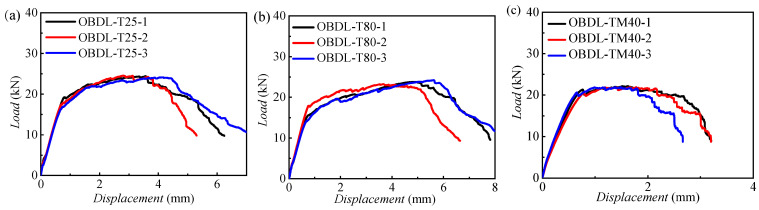
Load–displacement curves of CFRP–Al OBDL joints at three typical temperatures: (**a**) load–displacement curves during the tensile process of specimens at 25 °C; (**b**) load–displacement curves during the tensile process of specimens at 80 °C; (**c**) load–displacement curves during the tensile process of specimens at −40 °C.

Figure 6 presents the failure load–displacement curves for OBDL specimens at 25 °C, −40 °C, and 80 °C, with consistent experimental results. Initially, the load–displacement curves rise linearly. As displacement increases, the curve’s slope decreases, and the load rises gradually. At this stage, fiber breakage and matrix damage occur internally, yet the material can still bear loads. Further loading leads to multiple internal failures until the composite loses all load-bearing capacity and the load drops.

**Figure 7:**
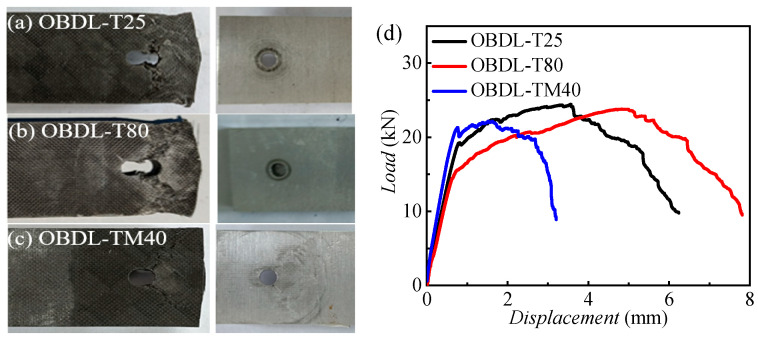
Tensile failure modes of OBDL joints at (**a**) 25 °C, (**b**) 80 °C, and (**c**) −40 °C; (**d**) load–displacement comparison curves of the three temperatures for OBDL joints.

Figure 7 shows the failure modes and load–displacement curves of CFRP–aluminum OBDL specimens at different temperatures. The failure modes and load–displacement curves for OBDL at room temperature in this paper are consistent with the patterns found in previous work [22]. From Figure 7a–c, the failure mode is mixed-mode. The regions around the bolt holes in the composite experience local bearing, with noticeable delamination damage and fan-shaped tensile failure on both sides of the holes, and fiber breakage is evident. The damage extent varies with temperature. In low-temperature environments, the compressive deformation around the bolt holes is smaller. The composite cross-section at the bolt hole along the width direction does not exhibit significant contraction. This is due to the resin matrix hardening in low-temperature conditions, which increases the composite’s compressive stiffness and strength compared to room temperature and high-temperature environments.

Figure 7d compares the load–displacement curves during the tensile process of specimens at different temperatures. The initial damage load is highest in low-temperature conditions, followed by room temperature, and lowest in high-temperature conditions. This is because the initial strength of the single-bolt double-shear connection depends on the composite’s compressive strength, which is greatest in low-temperature environments. As the displacement increases and the failure mode transitions from compressive deformation around the holes to tensile failure, the ultimate tensile strength of the connection is primarily determined by the composite’s tensile strength. The ultimate tensile strength is highest at room temperature, followed by high temperatures, and lowest at low temperatures. Additionally, the failure displacement of specimens in low-temperature conditions is much smaller than that in room temperature and high-temperature conditions.

### 3.3. Tensile Failure Results of OBSL and BBSL Specimens

**Figure 8:**
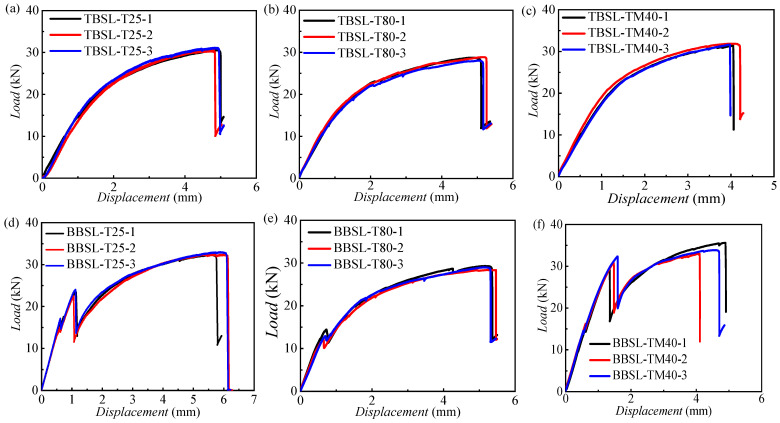
Load–displacement curves of TBSL and BBSL specimens at three typical temperatures: (**a**) load–displacement curves for one-bolt simple-lap (OBSL) at 25 °C; (**b**) load–displacement curves for one-bolt simple-lap (OBSL) at 80 °C; (**c**) load–displacement curves for one-bolt simple-lap (OBSL) at −40 °C; (**d**) load–displacement curves for hybrid bonded–bolted simple-lap (BBSL) joints at 25 °C; (**e**) load–displacement curves for hybrid bonded–bolted simple-lap (BBSL) joints at 80 °C; (**f**) load–displacement curves for hybrid bonded–bolted simple-lap (BBSL) joints at −40 °C.

**Figure 9:**
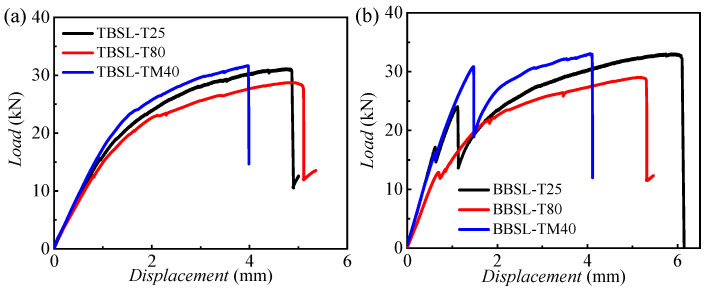
Load–displacement comparison curves of three temperatures for (**a**) TBSL and (**b**) BBSL joints.

Figure 8 shows the load–displacement curves for one-bolt simple-lap (OBSL) and hybrid bonded–bolted simple-lap (BBSL) joints under different temperatures, with three specimens per working condition. The results are consistent and repeatable. Figure 9 contrasts these curves at various temperatures.

For OBSL joints, the load–displacement curve initially exhibits a linear rise. Subsequently, the slope decreases due to progressive composite damage, resulting in non-linear behavior. This is a typical load curve corresponding to a pure bolted connection. Bolted joints initially exhibit linear elastic behavior, and then enter a non-linear softening region due to local bearing damage or plastic deformation of the bolts [3,4,5]. Following the peak load, a load drop occurs, indicating complete joint failure. As the temperature increases, both the initial stiffness and ultimate strength of these joints decrease. Specimens tested at room and low temperatures demonstrate higher ultimate strengths compared to those at high temperatures.

Unlike OBSL joints, BBSL joints exhibit one or more minor load drop events attributable to adhesive layer failure. As shown in Figure 9b, at both room and low temperatures, two such minor load drops occur during loading. The first drop signifies initial adhesive failure, while the second drop, accompanied by a significant decrease in slope, indicates widespread adhesive debonding. The load–displacement curves illustrate the typical characteristics of a hybrid joint that combines both adhesive bonding and bolt connections [9]. In the initial loading phase, the adhesive and the bolt work together to support the applied load as long as the adhesive remains intact. The hybrid joint initially exhibits a behavior similar to that of a pure adhesive joint, with a linearly increasing load–displacement curve. The first drop in load signifies the onset of adhesive failure. Subsequently, the load is primarily transferred to the bolt. In the final stage, the joint behaves like a typical bolted joint, with a progressive reduction in load-carrying capacity until the load resistance is completely lost.

**Figure 10:**
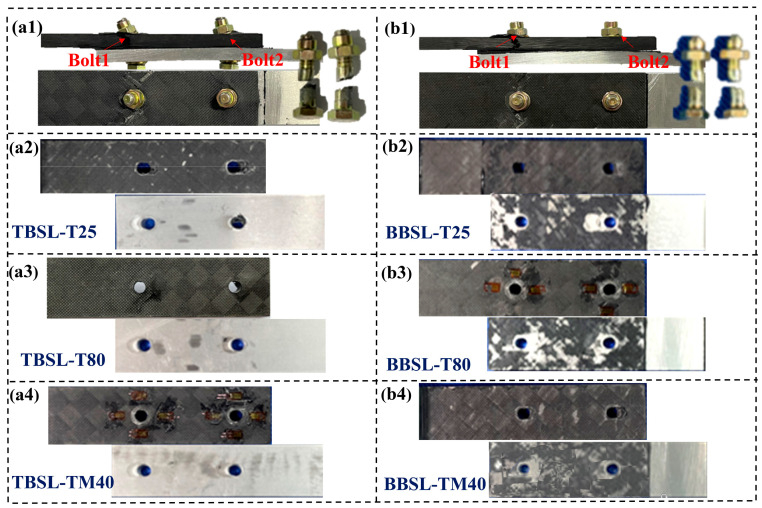
Failure modes of TBSL and BBSL joints at the three temperatures. (**a1**) Overall failure mode of TBSL. (**a2**–**a4**) Failure modes of TBSL at 25 °C, 80 °C, and −40 °C. (**b1**) Overall failure mode of the BBSL. (**b2**–**b4**) Failure modes of BBSL at 25 °C, 80 °C, and −40 °C.

Figure 10 shows the tensile failure modes of the TBSL and BBSL under different temperatures. It can be seen that the failure modes of the connection structure under three different temperatures are roughly the same. For the TBSL structure, Bolt1 near the end of the aluminum alloy showed obvious net-tension failure and local bearing failure around the hole. Bolt2 near the end of the CFRP laminates mainly showed a local extrusion failure around the hole, and the bolt underwent shear-out failure during the loading process. The failure modes of the BBSL connection structure are roughly the same as those of the pure bolt connection structure. Both are tensile failures of the sector-shaped section on both sides of the bolt holes of Bolt1 and compression failures caused by the bearing action on the right side of the bolts of Bolt1 and Bolt2. During the loading process, the failure mode of the adhesive layer in the BBSL connection structure at room and low temperatures is a mixed failure mode caused by both cohesion failure and interface failure. However, in the high-temperature environment, the failure mode of the adhesive layer mainly changes from the mixed failure mode to the interface failure, and the bonding force between the adhesive and the metal and composites decreases significantly. Under high-temperature conditions, the failure mode of the BBSL connection is almost the same as that of the TBSL connection due to the degradation of the adhesive performance.

### 3.4. Tensile Failure Results of TBDL and BBDL Specimens

**Figure 11:**
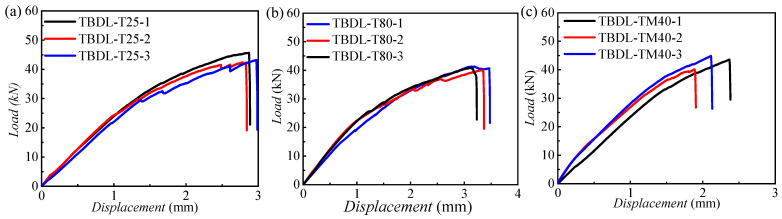

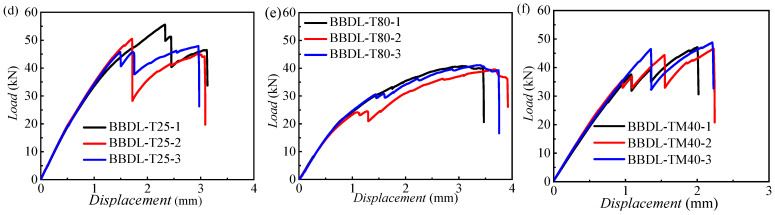
Load–displacement curves of TBDL and BBDL at three typical temperatures: (**a**) load–displacement curves for TBDL at 25 °C; (**b**) load–displacement curves for TBDL at 80 °C; (**c**) load–displacement curves for TBDL at −40 °C; (**d**) load–displacement curves for hybrid bolted–bonded (BBDL) joints at 25 °C; (**e**) load–displacement curves for hybrid bolted–bonded (BBDL) joints at 80 °C; (**f**) load–displacement curves for hybrid bolted–bonded (BBDL) joints at −40 °C.

**Figure 12:**
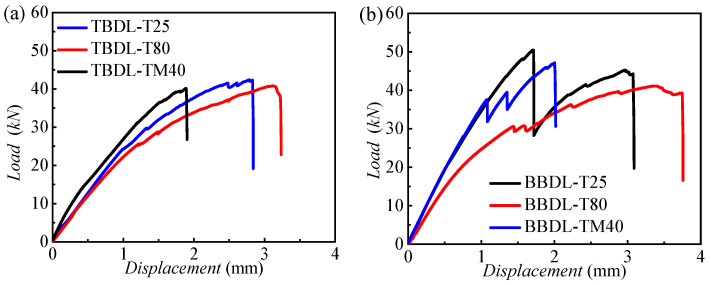
Load–displacement comparison curves of three temperatures for (**a**) TBDL and (**b**) BBDL joints.

Figure 11 presents the load–displacement curves for TBDL and hybrid bolted–bonded (BBDL) joints at 25 °C, −40 °C, and 80 °C, with three specimens per condition. Figure 12 compares these curves at the three temperatures.

For TBDL joints, the load–displacement curve initially increases linearly, then becomes non-linear due to progressive composite damage, eventually dropping at the ultimate load as the joint fails. Figure 12a shows that the initial stiffness of TBDL joints decreases with rising temperature, and failure occurs earlier at lower temperatures, as indicated by the smaller displacement at failure.

Unlike TBDL joints, BBDL joints exhibit one or more minor load drops during loading due to adhesive layer failure. Figure 12b indicates that the initial stiffness of hybrid joints also decreases with temperature. At high temperatures, the adhesive and resin matrix soften, reducing mechanical performance. Consequently, the BBDL joints at room and low temperatures show higher ultimate strength than those at high temperatures.

**Figure 13:**
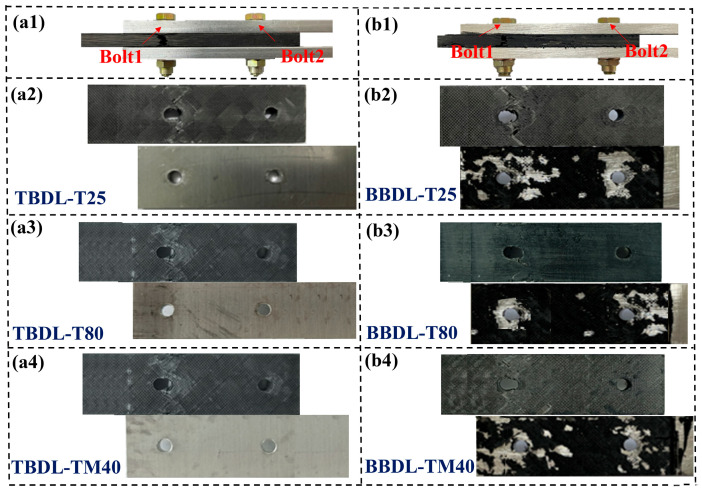
Failure modes of TBDL and BBDL joints at three temperatures. (**a1**) Overall failure mode of TBSL. (**a2**–**a4**) Failure modes of TBSL at 25 °C, 80 °C, and −40 °C. (**b1**) Overall failure mode of the BBDL. (**b2**–**b4**) Failure modes of BBDL at 25 °C, 80 °C, and −40 °C.

Figure 13 illustrates the tensile failure modes of TBDL and BBDL at 25 °C, −40 °C, and 80 °C. The failure modes are generally similar across temperatures. For TBDL joints, significant fan-shaped tensile failure and local bearing failure occur around Bolt1 near the aluminum alloy end. Extensive fiber breakage appears in the 45° plies on the upper and lower surfaces due to shear, while the middle 0° plies exhibit fiber breakage perpendicular to the loading direction, with cracks penetrating the specimen thickness. Around Bolt2 near the laminate end, minor local compressive failure is observed. Additionally, the compressive failure zone around Bolt1 shows delamination, matrix cracking, and fiber-matrix cracking.

The BBDL joints exhibit similar laminate failure modes to TBDL joints. However, at room and low temperatures, the adhesive layer in BBDL joints fails via a mixed mode of cohesive and interfacial failure. At high temperatures, the adhesive failure mode shifts to interfacial failure due to reduced adhesion between the adhesive and metal/composite, resulting in a lower bond strength.

## 4. Discussion

**Figure 14:**
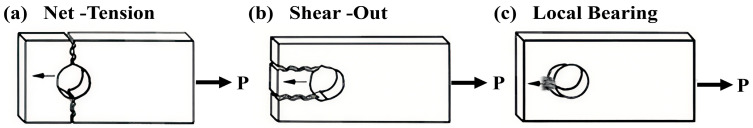
Typical failure modes of composite–metal joints: (**a**) net tensile, (**b**) shear-out, and (**c**) local bearing.

Below are several failure modes commonly seen in composite mechanical joints [27]: net-tension, shear-out, local bearing, cleavage, transverse splitting, and pull-out failures. Among them, cleavage, transverse splitting, and pull-out failures occur less frequently. They can be avoided through exquisite design. Net-tension and shear-out failures are the two primary brittle modes in multi-bolt joints, which occur suddenly and bring about catastrophic consequences. Local bearing failure, by contrast, is a damage mode that progresses slowly and gradually. Designers always try to achieve local bearing failure or a failure mode that incorporates it into structural connections. Figure 14 illustrates schematic diagrams of the three failure modes: net-tensile, shear-out, and local bearing.

**Figure 15:**
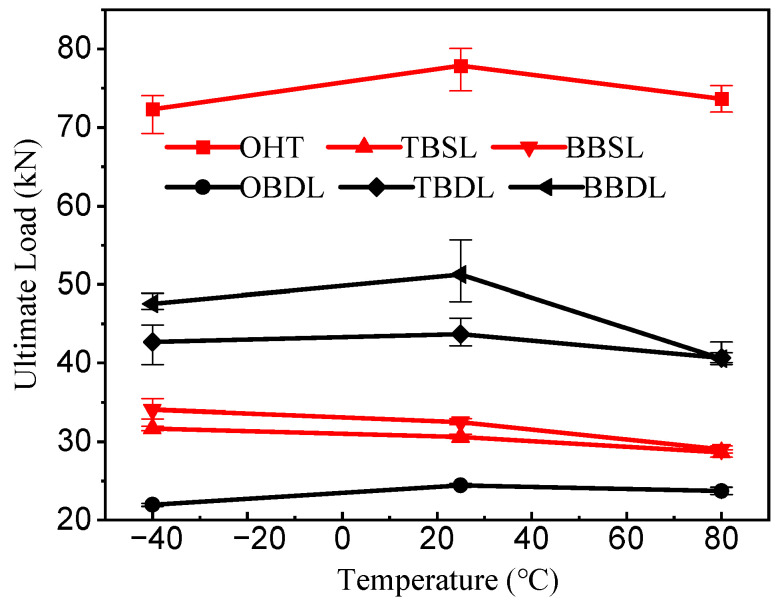
Ultimate load of OHT, TBSL, OBDL, BBSL, TBDL, and BBDL at three typical temperatures.

Figure 15 shows the ultimate load of the open-hole laminates and the CFRP–Al connection structure at different environmental temperatures. The maximum load and failure modes at different temperatures are also shown in Table 2. There is an obvious influence of temperature on the ultimate tensile load. Among them, under the working conditions of OHT, OBDL, TBDL, and BBDL, the ultimate tensile load of the specimens at room temperature is the largest, and at this time, tensile failure dominates the failure of the specimens. The ultimate tensile load is at −40 °C under the TBSL and BBSL conditions, at which point the dominant specimen is crushed by extrusion. Different connection structures determine the failure modes of the specimens under tensile loads.

**Figure 16:**
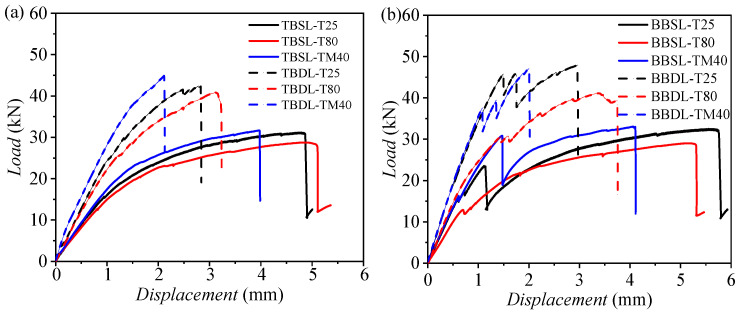
Load–displacement comparison curves at three temperatures for (**a**) TBSL and TBDL and (**b**) BBSL and BBDL joints.

Figure 16 presents the load–displacement curves for CFRP–Al connections, comparing the bearing capacities of single-lap and double-lap joints. In Figure 16a, for pure bolted joints, the single-lap joint (TBSL) has a lower initial stiffness than the double-lap joint (TBDL). At the ultimate load, the TBSL curve shows a significant load drop, leading to specimen failure. The ultimate loads of TBSL at temperatures of −40 °C, 25 °C, and 80 °C are 31.63, 31.06, and 28.29 kN, respectively, which are 29.5%, 26.20%, and 30.35% lower compared to the corresponding data of TBDL (44.85, 42.09, and 40.62 kN). The failure load for TBSL is lower than for TBDL, but the failure displacement is higher. Figure 16b shows that, for hybrid joints, the single-lap joint (BBSL) has a higher initial stiffness than the double-lap joint (BBDL). During loading, the BBSL curve shows minor load drops, followed by a major drop at the ultimate load. The BBSL failure loads are lower than those for BBDL. The ultimate loads for BBSL at temperatures of −40 °C, 25 °C, and 80 °C are 32.97, 32.10, and 28.66 kN, respectively, which are 19.8%, 31.66%, and 40.05% lower compared to the corresponding data for TBDL (47.80, 46.97, and 41.11 kN). The double-lap structure, with an extra metal plate, enhances the strength and stiffness. It reduces the concentration of stress in bolts, decreases the net tensile failure area, and mitigates adhesive failure.

**Figure 17:**
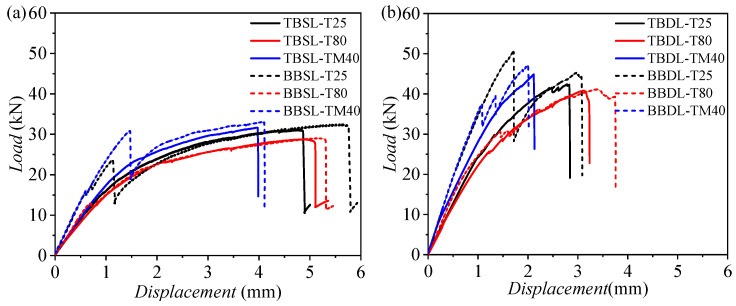
Load–displacement comparison curves at three temperatures for (**a**) TBSL and BBSL and (**b**) TBDL and BBDL joints.

Figure 17 shows the load–displacement curves for CFRP–aluminum connections, comparing the bearing capacities of bolted and hybrid joints. For single-lap joints (Figure 17a), the hybrid joint (BBSL) has a higher initial stiffness than the bolted joint (TBSL). The load–displacement curves exhibit significant temperature dependence. At −40 and 25 °C, the BBSL shows higher stiffness than the TBSL, but there is a significant load drop when the loading displacement is in the range of 1–2 mm. It then rises and converges with the stiffness of the TBSL, indicating that the bonding has failed at this point. The ultimate load of the BBSL is 32.93 and 32.27 kN at −40 and 25 °C, respectively, which is almost consistent with the final failure load of the TBSL (31.57 and 31.02 kN), but the failure displacement of the BBSL is higher. For 80 °C, the load–displacement curves of the BBSL and TBSL are almost consistent, indicating that the modulus of the adhesive is very low at high temperatures and is unable to provide shear-bearing capacity. In summary, the addition of bonding does not affect the ultimate tensile load for single-lap cases.

For double-lap joints, the ultimate load of the BBDL is 46.97 and 50.30 kN at −40 and 25 °C, respectively, which is significantly higher than the final failure loads of the TBDL (42.24 and 44.63 kN). This is because when the peak load occurs, the bonding has not completely failed, and it contributes to the improvement of the ultimate load of the connection. At 80 °C, the load–displacement curves of the BBDL and TBDL are almost consistent, indicating that the modulus of the adhesive is very low at high temperatures and is unable to provide shear-bearing capacity. In view of this, double-lap joints and adhesives with a high modulus at high temperatures are the optimal solution for improving the bearing capacity of the connection.

## 5. Conclusions

This study conducts an experimental investigation into the failure behaviors of CFRP–aluminum lap joints with various configurations under high- and low-temperature conditions. The following are the key conclusions:

Double-lap joints exhibit significantly greater strength compared to single-lap joints. The ultimate loads of the TBSL (single-lap joints) at temperatures of −40 °C and 25 °C are 29.5% and 26.20% lower, respectively, than those of the TBDL (double-lap joints). Similarly, the ultimate loads of the BBSL (hybrid single-lap joints) at −40 °C, 25 °C, and 80 °C are 19.8%, 31.66%, and 40.05% lower, respectively, compared to the corresponding data for the TBDL. Additionally, failure displacements of single-lap joints are higher than those of double-lap joints. Hybrid joints, which combine mechanical fasteners and adhesive bonding, demonstrate superior load-bearing capacity and higher initial stiffness compared to pure bolted joints. However, damage to the adhesive layer during loading can lead to load drops. At room and low temperatures, the ultimate loads of the BBDL (hybrid double-lap joints) are 46.97 kN at −40 °C and 50.30 kN at 25 °C, which are significantly higher than those of the TBDL (42.24 kN and 44.63 kN, respectively). Conversely, at high temperatures, the load–displacement curves of the BBDL and TBDL are almost consistent, indicating that the adhesive modulus is very low at elevated temperatures and unable to provide adequate shear-bearing capacity. Therefore, the development of double-lap joints and high-modulus adhesives for high-temperature applications is a promising direction for enhancing connection load-bearing capacity.

The failure modes of joints are influenced by both the connection configuration and the temperature environment. For single-lap joints (TBSL), the primary failure modes include net-tension failure, local bearing failure around the hole near the end of the aluminum alloy, and local extrusion failure around the hole near the end of the CFRP laminates, with the bolt undergoing shear-out failure during loading. The failure modes of bolted–bonded hybrid single-lap joints (BBSL) are similar to those of pure bolted connections. At room and low temperatures, adhesive layer failure in BBSL joints is characterized by a mixed mode of cohesive and interfacial failure. However, in high-temperature environments, the failure mode of the adhesive layer shifts predominantly to interfacial failure due to the significant reduction in bond strength between the adhesive and the metal/composite materials. These findings provide valuable references for the design of composite–metal connections in aircraft structures, especially in terms of understanding the effects of different joint configurations and temperature conditions on failure modes and load–bearing capacities.

## Figures and Tables

**Table 1:** List of working conditions.

Specimen Name	Connection Type	Temperature	Label	Specimen Number
OHT	Open-hole, No Joint	RT −40 °C 80 °C	OHT-T25 OHT-TM40 OHT-T80	3
OBDL	One-bolt, Double-Lap	RT −40 °C 80 °C	OBDL-T25 OBDLTM40 OBDL-T80	3
TBSL	Two-bolt, Single-Lap	RT −40 °C 80 °C	TBSL-T25 TBSL-TM40 TBSL-T80	3
TBDL	Two-bolt, Double-Lap	RT −40 °C 80 °C	TBDL-T25 TBDL-TM40 TBDL-T80	3
BBSL	Bonded–bolted hybrid, Single-Lap	RT −40 °C 80 °C	BBSL-T25 BBSL-TM40 BBSL-T80	3
BBDL	Bonded–Bolted hybrid, Double-Lap	RT −40 °C 80 °C	BBDL-T25 BBDL-TM40 BBDL-T80	3

**Table 2:** Summary of specimen failure loads and failure modes.

Label	Ultimate Load (kN)	Failure Mode
OHT-TM40	72.33	Net-Tension
OHT-T25	77.89	Net-Tension
OHT-T80	73.66	Net-Tension
OBDL-TM40	21.96	Net-Tension + Local Bearing + Shear-out
OBDL-T25	24.41	Net-Tension + Local Bearing + Shear-out
OBDL-T80	23.70	Net-Tension + Local Bearing + Shear-out
TBSL-TM40	31.68	Net-Tension + Local Bearing
TBSL-T25	30.59	Net-Tension + Local Bearing
TBSL-T80	28.66	Net-Tension + Local Bearing
TBDL-TM40	42.70	Net-Tension + Local Bearing
TBDL-T25	43.69	Net-Tension + Local Bearing
TBDL-T80	40.67	Net-Tension + Local Bearing
BBSL-TM40	34.10	Net-Tension + Local Bearing + Shear-out
BBSL-T25	32.50	Net-Tension + Local Bearing + Shear-out
BBSL-T80	29.00	Net-Tension + Local Bearing + Shear-out
BBDL-TM40	47.58	Net-Tension + Local Bearing + Shear-out
BBDL-T25	51.27	Net-Tension + Local Bearing + Shear-out
BBDL-T80	40.56	Net-Tension + Local Bearing + Shear-out

## Data Availability

The original contributions presented in this study are included in the article. Further inquiries can be directed to the corresponding author.
